# Role of mechanical hysteroscopic tissue removal system in post-molar gestational trophoblastic neoplasia management: case report and literature review

**DOI:** 10.1093/jscr/rjaf190

**Published:** 2025-04-06

**Authors:** Sophie Schoenen, Katty Delbecque, Frédéric Kridelka, Florence Hanocq, Frédéric Goffin, Patricia Nervo

**Affiliations:** Département de Gynécologie-Obstétrique, Hôpital Universitaire de Liège, Université de Liège (ULiège), Avenue de L'Hôpital 1, 4000 Liège, Belgique; Département d'Anatomie Pathologique, Hôpital Universitaire de Liège, Université de Liège (ULiège), Avenue de L'Hôpital 1, 4000 Liège, Belgique; Département de Gynécologie-Obstétrique, Hôpital Universitaire de Liège, Université de Liège (ULiège), Avenue de L'Hôpital 1, 4000 Liège, Belgique; Département de Gynécologie-Obstétrique, Hôpital Universitaire de Liège, Université de Liège (ULiège), Avenue de L'Hôpital 1, 4000 Liège, Belgique; Département de Gynécologie-Obstétrique, Hôpital Universitaire de Liège, Université de Liège (ULiège), Avenue de L'Hôpital 1, 4000 Liège, Belgique; Département de Gynécologie-Obstétrique, Hôpital Universitaire de Liège, Université de Liège (ULiège), Avenue de L'Hôpital 1, 4000 Liège, Belgique

**Keywords:** hydatidiform moles, gestational trophoblastic neoplasia, hysteroscopic resection

## Abstract

Complete hydatidiform moles are precancerous gestational trophoblastic diseases, with 15%–20% progressing to post-molar neoplasia. The gold standard treatment of low-risk neoplasia is single-agent chemotherapy. A second curettage is an alternative to avoid chemotherapy; hysteroscopic resection is not described in the guidelines. We report a case of post-molar neoplasia treated using a mechanical hysteroscopic tissue removal system (TruClear°) and review the limited literature on the subject. A patient was diagnosed with low-risk post-molar neoplasia in our department. Ultrasound evaluation revealed uterine trophoblastic retention, which was managed with mechanical hysteroscopic resection. In conclusion, hysteroscopic resection could be a potential alternative to second curettage in highly selected cases of low-risk post-molar neoplasia.

## Introduction

Gestational trophoblastic diseases (GTD) encompass a spectrum of rare placental pathologies [1]. Premalignant forms include partial and complete hydatidiform moles, while malignant forms, called gestational trophoblastic neoplasia (GTN), include invasive moles, gestational choriocarcinoma, placental site trophoblastic tumors, and epithelioid trophoblastic tumors. Post-molar GTN is diagnosed based on International Federation of Gynecology and Obstetrics (FIGO) biological criteria, which assess human chorionic gonadotropin (hCG) kinetics, or through histological analysis. Between 15% and 20% of complete moles progress to post-molar GTN, characterized by persistently elevated hCG levels, with only 3% of these cases developing into choriocarcinoma [2].

The gold standard treatment of GTN is chemotherapy, with the regimen determined by the tumor’s risk profile. The FIGO/World Health Organization (WHO) scoring system, based on eight predictive factors, is used to assess the risk of resistance to single-agent chemotherapy. Low-risk tumors, defined by a risk score of 6 or below, are treated with single agent chemotherapy [1].

Second curettage and hysterectomy are surgical alternatives to avoid chemotherapy [1, 3]. However, hysteroscopic resection is not a standard treatment for GTN. To date, only one case of low-risk post-molar neoplasia treated with hysteroscopy has been reported, and the patient required subsequent chemotherapy [4]. The emergence of mechanical hysteroscopic tissue removal systems has enabled outpatient hysteroscopic procedures, but their use in GTD management has never been described.

## Case report

We report the case of a 34-year-old pregnant patient treated in our Department of Obstetrics and Gynecology at the University Hospital. At 8 weeks of amenorrhea, a hydatidiform mole was suspected based on ultrasound findings and an elevated serum hCG level (220 000 IU/L). The patient was completely asymptomatic. Ultrasound-guided suction curettage was performed. Histopathological result confirmed a complete mole. Follow-up with hCG monitoring was done weekly.

One month later, post-molar GTN was diagnosed based on the 2000 FIGO criteria, as hCG levels had increased by ˃10% in three consecutive measurements over 2 weeks. After imaging work-up, the patient was assigned a FIGO/WHO risk score of 1, indicating a low-risk disease with minimal risk of resistance to single-agent chemotherapy.

**Figure 1:**
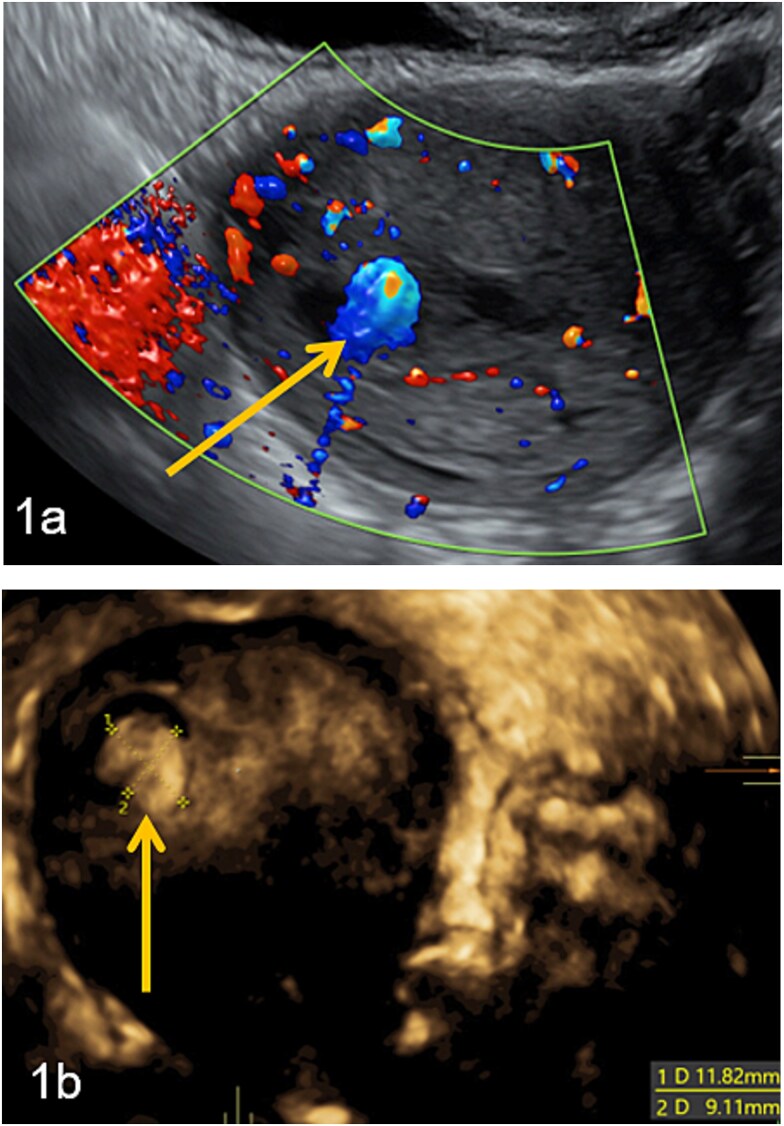
Intra-uterine vascularized mass (arrows) diagnosed by Doppler (a) and 3D-pelvic (b) ultrasound. The Doppler color score is 4 and there is no suspicion of myometrial invasion (continuous halo surrounding the lesion).

Doppler and 3D-pelvic ultrasound revealed a 12 × 9 mm vascularized intrauterine mass with a Doppler color score of 4, with no signs of myometrial invasion (Fig. 1). Two treatment options were proposed to the patient: initiation of single agent chemotherapy with methotrexate or surgical removal of the retained uterine tissue using a mechanical hysteroscopic tissue removal system.

**Figure 2:**
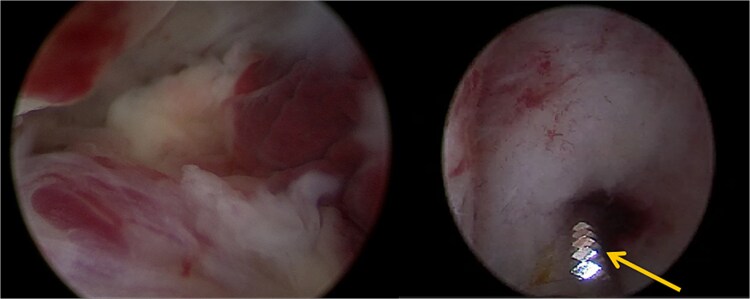
A trophoblastic resection by hysteroscopic tissue removal system TruClear° was performed with the soft tissue shaver (arrow).

The patient opted for the latter. The procedure was performed in the operating room under spinal anesthesia due to the risk of hemorrhage. Using the TruClear System° (Medtronic) with saline irrigation, the remaining trophoblastic tissue was visualized and resected under direct hysteroscopic control. The soft tissue shaver was used with an oscillation speed of 1500 cycles per minute (Fig. 2). The procedure lasted 15 min, with no significant fluid absorption. The patient was discharged the same day without complications or significant bleeding. Histopathological analysis confirmed retained complete hydatidiform mole. hCG monitoring demonstrated normalization within 3 weeks following the procedure. Monthly hCG monitoring was then carried out for a period of 1 year while the patient was using effective contraception. Informed consent was obtained from the patient for the publication of this case.

## Discussion

The gold standard treatment for GTN is chemotherapy. The curative effect of a second uterine evacuation by curettage has been debated in the literature, with reported cure rates ranging from 9.4% to 83% [5–9]. A recent meta-analysis demonstrated that a second curettage offers a significant advantage in avoiding chemotherapy and reducing the number of chemotherapy courses in cases of persistent trophoblastic diseases [10].

Although several studies have reported the role of hysteroscopy in the diagnosis of GTD and GTN [11, 12], the available literature on hysteroscopic management of GTD remains scarce. A series of 36 cases of hydatidiform moles treated by endoscopic management instead of conventional dilatation and curettage has been reported [13]. In this study, three patients were diagnosed with GTN, all of whom were treated with chemotherapy alone. To our knowledge, only one case of GTN treated with conventional hysteroscopic resection has been reported [4]. Histological analysis confirmed the retention of a hydatidiform mole, and three courses of single-agent chemotherapy were required after surgery.

Hysteroscopic resection allows for the removal of tissue under visual control. A randomized study showed that retained products of conception were more completely removed by hysteroscopic morcellation than by vacuum aspiration curettage [14].

New hysteroscopic technologies enable tissue removal without the use of energy. For the treatment of benign pathologies (endometrial polyps and submucosa myomas), a meta-analysis showed that hysteroscopic tissue removal systems are more efficient, safer, and significantly reduce operative time compared to conventional hysteroscopy [15]. In this case, the patient underwent hysteroscopic removal using a 6-mm TruClear° hysteroscopic tissue removal system (Medtronic Parkway, Minneapolis, MN), which allowed for visually controlled resection of trophoblastic tissue.

In our opinion, a second uterine evacuation should be performed in cases of post-molar low-risk GTN with suspected uterine remnants. Mechanical hysteroscopic management allows for the resection of retained trophoblastic tissue under direct visual control without the use of energy. This technique should be considered in selected cases of retained molar tissue, provided there is no suspicion of myometrial invasion or distant metastases. The aim of surgery is to remove all macroscopic disease, but microscopic trophoblastic cells may remain. Therefore, biological follow-up with hCG level monitoring is essential. In case of low-risk disease, monthly hCG monitoring is recommended for 1 year, regardless of whether the treatment was surgical or medical.

## Conclusion

A second uterine evacuation should be considered in selected cases of post-molar neoplasia to avoid unnecessary chemotherapy. Mechanical hysteroscopic resection is a feasible technique that enables complete tissue removal without the use of energy.
